# Fluorescence microscopy image noise reduction using a stochastically-connected random field model

**DOI:** 10.1038/srep20640

**Published:** 2016-02-17

**Authors:** S. A. Haider, A. Cameron, P. Siva, D. Lui, M. J. Shafiee, A. Boroomand, N. Haider, A. Wong

**Affiliations:** 1 Vision and Image Processing (VIP) Research Group, Department of Systems Design Engineering, University of Waterloo, 200 University Avenue W, Waterloo, ON, N2L 3G1, Canada; 2 Department of Medical Biophysics, University of Toronto, 610 University Avenue, Toronto, ON, M5G 2M9, Canada

## Abstract

Fluorescence microscopy is an essential part of a biologist’s toolkit, allowing assaying of many parameters like subcellular localization of proteins, changes in cytoskeletal dynamics, protein-protein interactions, and the concentration of specific cellular ions. A fundamental challenge with using fluorescence microscopy is the presence of noise. This study introduces a novel approach to reducing noise in fluorescence microscopy images. The noise reduction problem is posed as a Maximum A Posteriori estimation problem, and solved using a novel random field model called stochastically-connected random field (SRF), which combines random graph and field theory. Experimental results using synthetic and real fluorescence microscopy data show the proposed approach achieving strong noise reduction performance when compared to several other noise reduction algorithms, using quantitative metrics. The proposed SRF approach was able to achieve strong performance in terms of signal-to-noise ratio in the synthetic results, high signal to noise ratio and contrast to noise ratio in the real fluorescence microscopy data results, and was able to maintain cell structure and subtle details while reducing background and intra-cellular noise.

Since its conception, fluorescence microscopy has become an essential part of a molecular biologist’s toolkit. It allows for the assaying of a multitude of parameters such as subcellular localization of proteins, changes in cytoskeletal dynamics, protein-protein interactions and the concentration of specific cellular ions. This technique utilizes fluorescent molecules or “fluorophores”. The principle is that after a fluorophore absorbs or is excited by a photon of a particular wavelength, it fluoresces by releasing another photon, typically of a longer wavelength. The difference between the excitation and emission wavelengths is known as the Stokes shift. This property allows for a fluorescence microscope with a specific wavelength illuminant to only view light emitted by the fluorophore.

Fluorescence microscopy provides an insight into the cellular world, but due to the inherent characteristics of the modality, non-representative intensity variations are present in the images. The variations in these images can interfere with the biologist’s research by making it difficult to observe low intensity signals and fine detail. These variations primarily exists due to the Poisson statistics of the incoming photons onto the detector with additional influence from sources like the detector itself, the optical setup, and the experimental parameters^1^,^2^. In addition, there can be additional unintentional signal from auto-fluorescence within cells and from the unintended accumulation of fluorescent tags on organelles producing a signal bias which can interfere with observations^3^.

To reduce the intensity variations that can come from the detector, some fluorescence microscopes use an electron multiplying charge-coupled detector^4^. This detector uses an electron multiplying register at the end of the normal serial register to amplify weak signals before noise is added by the readout amplifier^5^. While this method does reduce the effects of readout noise, the resulting images still exhibit variations due to photon arrival and thus post-processing algorithms are needed to reduce the variations further. A number of algorithms have been proposed for the purpose of noise reduction, ranging from wavelet^6^,^7^,^8^,^9^, iterative^10^ and diffusion^11^,^12^,^13^,^14^,^15^ methods to non-local mean^16^,^17^ and graphical model frameworks^18^,^19^,^20^ based methods, all of which make use of the underlying characteristics of the images.

In many imaging applications, the typical assumption is that the imagery is contaminated by an additive, stationary Gaussian white noise source, and as such traditional noise reduction algorithms based on stationary Gaussian noise assumptions may be utilized. However, in the case of fluorescence microscopy, the imagery is contaminated with intensity variation and noise from inherent properties of the imaging modality and capturing apparatuses. The variation from the imaging modality is dominated by photon arrival and can be modelled using Poisson statisitcs, while the noise from the capturing apparatuses can be modelled from Gaussian statistics^2^,^21^,^22^. Due to the variations (which will be here on referred to as noise, for simplicity) from the imaging modality following a different statisitcal model, the direct applciation of noise reduction models based on stationaty Gaussian noise assumptions would result in sub-optimal performance and possibly the rise of processing artefacts. Existing noise reduction algorithms accommodate for Poisson and Poisson-Gaussian noise sources through several strategies. Variance stabilisation transforms^9^,^17^,^23^ have been used to transform Poisson-Gaussian noise into signal-independent, constant variance additive Gaussian noise^24^,^25^, allowing it to be handled using traditional methods based on stationary Gaussian noise assumptions. Risk estimators for noise parameter estimation that incorporates Poisson parameters directly into their formulation have also been used^7^,^10^,^18^,^26^. Lastly, algorithms have been proposed that can take the stochastic nature of the Poisson noise into account and model distribution similarly on stochastic distances^16^. All these methods perform fluorescence microscopy noise reduction to good effect.

In this paper, we propose a novel algorithm for noise reduction of fluorescence microscopy images. We formulate the noise reduction problem as a maximum a posteriori (MAP) problem that utilizes a novel random field model called a stochastically-connected random field (SRF) model. This model better accounts for abrupt data uncertainties while preserving structure making it well-suited for dealing with the fine detail and noise in fluorescent microscopy images and is demonstrated doing so in this paper with competitive quantitative results.

The rest of the paper is organized as follows. The formulation of the stochastically-connected random field model and its application to noise reduction of fluorescence microscopy images will be shown in sec:method along with the choice of parameters for the proposed approach and the experimental design for validation will be described. Finally, experimental results are presented in sec:results, with discussion presented in sec:discussion.

## Methods

In the fluorescence microscopy noise reduction problem, the underlying goal is to obtain an estimate of a noise-free fluorescence microscopy image, 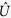, given noisy observations, 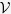, of the noise-free image, *U*. In the case of fluorescence microscopy, *U* will be analogous to a representation of the number of true incident photons on the detector. We define *U*, 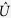, and 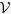 as sets of *M* pixels:


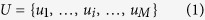



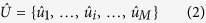



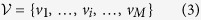


The noise-free image, *U*, has been contaminated by noise processes following a combination of Poisson and Gaussian statistics due to the inherent properties of the imaging modality and capturing apparatuses^2^,^21^,^22^:


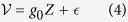


where *Z* is *U* degraded by a Poisson process, *g*_0_ is the gain applied to *Z*, and 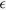 is a Gaussian distribution, 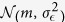.

We formulate this noise reduction problem as a MAP estimation problem. Given 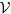, we obtain the MAP estimate 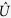 as:


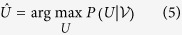


where 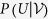 is the posterior probability. Taking a note from conditional random fields [?], we can reformulate 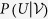 by decoupling it into the product of a unary term and a pairwise term:


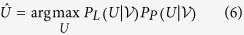


where 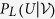 is the unary term and 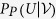 is the pairwise term of the posterior probability 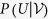. In the case of noise reduction, the pairwise term is a spatial constraint that will enforce a local smoothness prior on *U* based on the observations 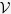, while the unary term seeks to minimize the error between the noise-free estimate at a pixel (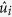) and the observation (*v*_*i*_). The conditional independence assumption of the measurements given their states^27^ gives us the general noise reducing form of the MAP estimate as:


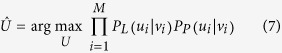


Modelling of the pairwise term in a way that provides a meaningful constraint that accounts for abrupt data uncertainties while maintaining awareness of image structure is important for noise reduction applications like fluorescence microscopy, where fine structure is important. In fluorescence microscopy, fluorophores can be used to not only highlight the relatively large cytoplasmic fluid, but also highlight fine protein dynamics. These fine protein dynamics can be lost depending on the choice of the pairwise term. To this end, we propose a novel type of random field (RF), which we will denote as a stochastically-connected random field (SRF), to model the pairwise term 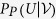. We first define the SRF model, then formulate our noise reduction problem using an SRF model.

### Stochastically-connected Random Field (SRF)

**Figure 1:**
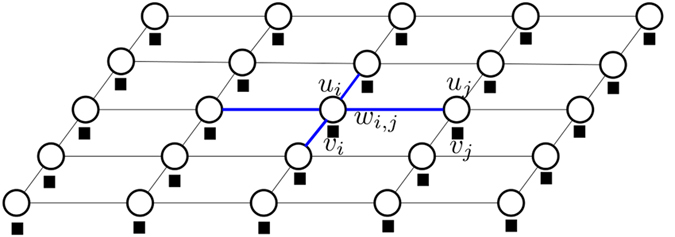
A random field with 4-connected smoothness constraint. Each connection weight *w*_*i*,*j*∈*c*_ is obtained as an exponentially weighted difference between observations *u*_*i*_ and *u*_*j*_.

An example of a popular random field (RF) configuration is illustrated in Fig. 1. Each node in the RF represents a pixel in the image, and the random variables *u*_*i*_ and *u*_*j*_ are the noise-free image intensities of the *i*^*th*^ and *j*^*th*^ nodes. The pairwise term based upon the RF can be written as:


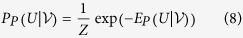



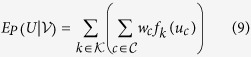


where *Z* is a normalization constant to represent the value as probabilities, 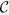 denotes the set of all clique templates in the RF, *u*_*c*_ is the subset of states of the RF determined by clique templates 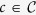, *f*_*k*_ denotes the *k*^*th*^ arbitrary feature function, and 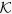 denotes the number of feature functions.

In a standard RF, *u*_*i*_ is connected to all sites in a local neighbourhood with a weight *w*_*i*,*j*_ as illustrated in Fig. 1 (since we use a binary clique in this paper, we change *w*_*c*_ to *w*_*i*,*j*_ for simplicity of formulation). For example in Fig. 1, an 8-connected neighbourhood structure is illustrated. A common approach to obtaining weights for the RF from the observations is:


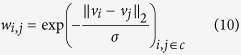


where 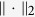 is the *L*_2_ norm and *σ* is a smoothness constant. There are two main limitations with this approach. First, it is sensitive to abrupt data uncertainties due to factors such as outlier intensities, which will still have a contribution to the smoothness term and thus affect estimation quality. Second, it has poor sensitivity to structural characteristics and detail in images, thereby also potentially reducing estimation quality. Both of these limitations can affect noise reduction performance if used for the purpose of fluorescence microscopy. Some approaches have tried to improve outlier robustness of such weights [?], but addressing the second main limitation at the same time as the first main limitation is not well-explored.

To address these two important issues, one can enforce a deterministic threshold on the weights based on some similarity criteria such as the *L*_2_ norm (similar to the approach taken by Boykov *et al.*^28^):


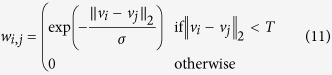


where *T* is a threshold that dictates edge connectivity. In the presence of an abrupt change 
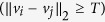
, the connectivity weight *w*_*i*,*j*_ becomes 0, thus indicating a lack of connectivity between sites *i* and *j* and hence no smoothness constraint is enforced between these two sites. Therefore, the connectivity of sites in the RF is enforced deterministically based on abrupt changes in the underlying data characteristics. In this way, one can improve robustness to abrupt data uncertainties and improve structural preservation. One of the biggest issues with taking such an approach is that it is highly sensitive to the choice of the connectivity threshold *T*. A high *T* may improve structural preservation but be poor at handling noise and data uncertainties. A low *T* may provide better noise and data uncertainty but lead to poor structural preservation. To address this issue, we avoid this “hard thresholding” approach by employing random graph theory^29^,^30^, where edge connectivity between sites is determined in a stochastic manner. This amalgamation of random graph theory and random field theory results in an SRF.

The SRF is an RF where the edge connectivity and weights are stochastically determined, resulting in random graphs where the edges exist with a certain probability *γ*. The clique (clq) structure and connectivity of two nodes are determined based on a distribution (illustrated here using indicator function 1(⋅)):


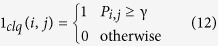



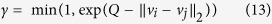


where *Q* is a flexibility constant and *P*_*i*,*j*_ is the probability of connectivity between *i* and *j*. As such, the weighting function (weight of the smoothness constraint) for SRF can be written as:


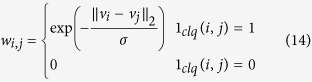


The novelty of this random field relates to the stochastic clique structures. The set of clique structures of the SRF, 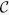, is determined based on a distribution. In this paper, binary cliques are utilized; according to the neighborhood 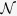, two nodes 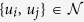 are connected based upon a probability drawn from the distribution.

**Figure 2:**
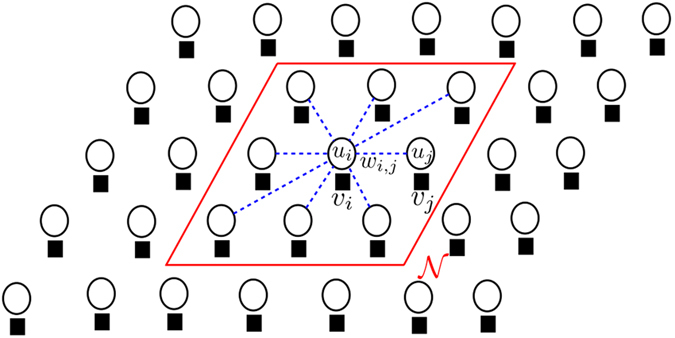
A stochastically-connected random field (SRF) with an 8-connected smoothness constraint. Each connection weight *w*_*i*,*j*∈*c*_ is stochastically sampled from an exponential distribution. The use of stochastic weights results in an uncertainty on which of the weighted edges between sites exist, as indicated by the dashed connections.

The connectivity weights for edges between sites, as well as the existence of said edges, are no longer deterministic but randomly sampled from an exponential distribution, as determined by *γ* and illustrated by the dashed connections in Fig. 2. The exponential distribution does allow for the connection of pixels across more abrupt data changes with a small probability. The energy function for SRF is the same as Eq. (9) with weights defined per Eq. (14).

To compute the unary term (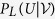), we assume the conditional independence assumption of measurement given label. Due to the Poisson-Gaussian characteristic noise sources in fluorescence microscopy (as modelled in Eq. (4)), we wish to account for the Poisson-Gaussian noise characteristics in the unary term 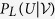. One challenge with incorporating the Poisson-Gaussian noise statistics in the unary term directly is that the optimization process comes significantly more challenging from a computational perspective. Therefore, to reduce the complexity of the optimization process while still taking the Poisson-Gaussian noise characteristics into account, a variance-stabilization transform^9^,^22^,^24^,^25^,^31^,^32^ is incorporated into an energy function 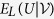, which in effect transforms the Poisson-Gaussian noise characteristics into approximately Gaussian distributed noise characteristics:


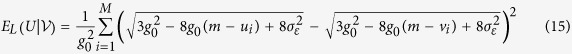


where *g*_0_ is the gain on the photon signal, *m* and *σ*_*ε*_ are parameters for the estimated Gaussian noise distribution 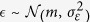. This energy function 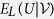 can then be used within a negative exponential unary term 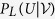 as:
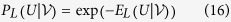


To minimize the energy function and infer the configuration with highest probability, the unary and pairwise energy functions (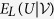 and *E*_*P*_(*U*)) are aggregated:


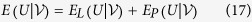


Next, we explain noise reduction using SRF.

### Noise reduction with SRF

**Figure 3:**
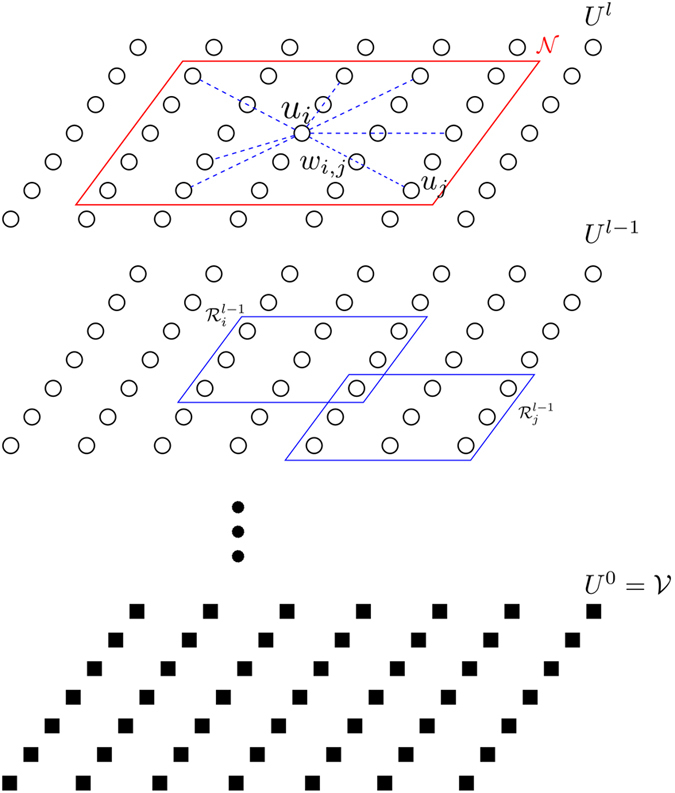
A multi-layer, high-order, stochastically-connected random field (SRF). At each layer, the connection weights 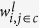 are stochastically obtained from the exponential difference between the *L*2 norm of the regions 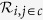 surrounding each of the pairs of points from the previous solution *U*^*l* − 1^ and thusly, each node is stochastically-connected to a local neighbourhood 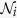, where size of 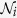 is greater than 3 × 3, creating higher-order connections.

We use a multi-layer, higher order SRF for the noise reduction of fluorescence microscopy images. A higher order connectivity model was employed to better characterize local spatial-feature context, while the multi-layer structure allows for better characterization of complex structural data intricacies at different scales. The multi-layer structure is inspired by iterative scale-space, with the output state of layer *l* − 1 being used as the observation of layer *l*. The multi-layer, higher order SRF is illustrated in Fig. 3. The pairwise energy for the *l*^th^ layer SRF can be written as:


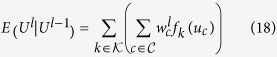


where *U*^*l*^ is the state at layer *l*, *U*^*l* − 1^ is the state at layer *l* − 1, and 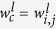 is the weight of the smoothness constraint between pixels *i* and *j* computed using *U*^*l* − 1^.


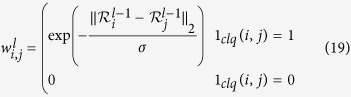


The 1_*clq*_(*i*, *j*) has the same functionality like before but:


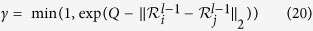


where 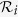 is region defined by a vector of observation values from *U*^*l* − 1^ corresponding to a set of nodes centered at node *i*, and 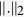 is the L2-norm between two vectors. Unlike Eq. (14), the computation of 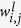 in Eq. (19) uses the solution from the previous level as well as 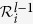 and 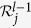 which are regions surrounding 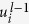 and 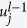. This is because the regional difference allows for characterization of local spatial-feature context and thus, results in better estimation of intensity differences in the presence of noise rather than using per-pixel differences. Typically, this assumption holds for fluorescence microscopy due to the point spread function of the imaging system being larger than a single pixel and having a Gaussian profile^33^.

The multi-layer structure allows us to compute our smoothness weight 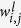 based on the solution of the previous layer. Not only does it allow for better characterization of data intricacies at different scales, but this also results in an iterative improvement on the smoothness as the noise is reduced at each layer, allowing a better estimate of the weight to be computed.

The edges stochastically connect *i* to all sites *j* in a local neighbourhood 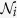 surrounding *i*, where the size of 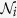 is greater than or equal to 3 × 3. This results in a higher order SRF, but we find that the higher-order connections are particularly good for fluorescence microscopy images due to the presence of large homogeneous regions in the images.

By utilizing this novel clique structure and its corresponding weights, the SRF can involve the measurement into the priori model implicitly. As a result, the states in the SRF are not assumed independent on measurements.

### Optimisation

We obtain an approximate solution of Eq. (9), using Eq. (18), by using gradient descent. The iterative solution for a site *s* is:


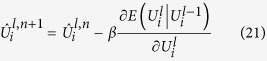


where *β* is the step size and *n* = 0…*N* − 1 is the *n*^*th*^ iteration of gradient descent for a total of *N* iterations and we introduce *λ* as a regularization term between the likelihood and prior term for our MAP problem. We initialise the iterative solver with the noisy image 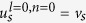 and at each layer *l* > 1, we set 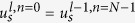.

For each layer *l*, we do not completely solve for 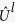, instead only taking a single gradient step (i.e. *N* = 1). First we fix 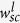 then solve for 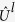, then we fix 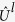 and solve for 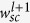. In this way we can simultaneously optimise for *U* and *w*_*sc*_ for our multi-layer SRF.

### Algorithm Design

The choice of parameters is important when using an SRF model to address the fluorescence microscopy noise reduction problem. Several parameters can be tuned to produce varying estimates of the spatial prior in the SRF. In summary, there are two parameters of the SRF whose consequences are worth mentioning: i) the neighbourhood size, 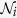 at site *i* and ii) the flexibility constant, *Q*. The modification of 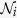 at site *i* dictates the spatial extent to which we can guess smoothness. The modification of the flexibility constant, *Q*, adjusts the existence of edges between states where there is likely to be a smooth signal. We will fix 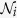 to have a fairly large size as recommended in this paper, 11 × 11, and the flexibility constant will be the smoothness constant. The step size parameter for gradient descent is trivial and will be fixed to *β* = 0.5.

The remaining parameters for noise reduction (ie. the number of layers, *l*, and the regularization term, *λ*) will be tuned using quantitative metrics. Explanation for the experimental setup for the parameter tuning will be explained in section 0. The smoothness constant, *σ*, for each channel was chosen using estimates of the background SNR.

## Experimental Setup

In this section we outline the synthetic and empirical datasets used and the quantitative metrics used in experimenting and validating the performance of the proposed algorithm (SRF) existing methods.

### Synthetic Data

To objectively demonstrate the efficacy of the SRF on reducing noise in fluorescence microscopy images, we generated a synthetic fluorescence microscope image. To mimic the formation of an image from a fluorescence microscope, we took the general structure of an image from the Yeast Resource Centre Public Image Repository^4^ and incorporated additional information from different sources, contributing to both signal and noise.

The synthetic image is based on the cyan channel of experiment 24 from the Yeast Resource Centre Public Image Repository(YRCPIR)^4^, where we had its noise floor removed and then quantized on an 8-bit scale. These new pixel intensities were then interpreted as the number of photons captured without statistical uncertainties (*U*).

**Figure 4:**
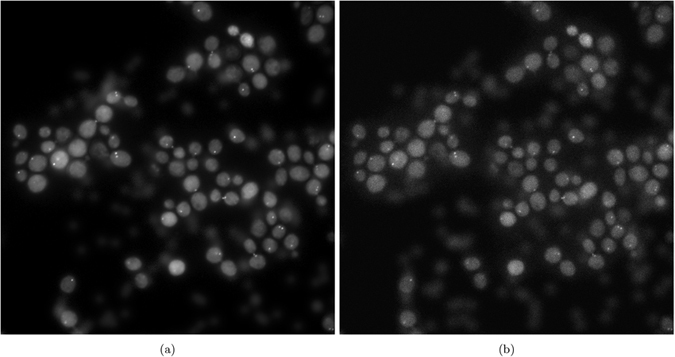
Synthetic noise phantoms. (**a**) Noise-free phantom, and (**b**) Noise contaminated phantom.

Similar to Boulanger *et al.*^22^, we added additional background signal that can arise from autofluorescence within the cell as well as from the unintended accumulation of fluorescent tags on organelles. We simulated this background signal with 200 Gaussian profiles with an assumed photon count from 0 to 20. To simulate the statistical uncertainty associated with photon arrival, realized as a Poisson distribution, we followed Verveer *et al.*^3^ by using a scaled version of the certain photon count, *βU*, as the mean of a Poisson distribution. With an increase in *β*, the average number of photons detected increases and decreases noise level. Verveer *et al.*^3^ describe the reciprocal of *β* as the photon-conversion factor which is the product of several multiplicative factors, including integration time and the quantum efficiency of the detector. The dark current from the detector that arises from thermal energy can be modelled as additive Gaussian noise to the signal on the detector. We added Gaussian noise with mean of 12 and a standard deviation of 0.5. Figure 4 shows the FM phantom with no added noise and the phantom with added noise. The phantom with added noise will be the baseline measure of noise reduction in the synthetic tests.

We generated images with varying *β* parameters to simulate conditions ranging from photon starved (*β* = 0.1) to photon nominal (*β* = 0.9). All the other added signal and noise stayed constant to mimic constant temperature and consistent system specifications.

### Empirical Data

Raw fluorescence microscopy datasets were taken from YRCPIR^4^ for algorithm validation. These images were taken by members of the Trish Davis laboratory in the Department of Biochemistry at the University of Washington studying *Saccharomyces cerevisiae*. The images were of live cells mounted on agarose pads acquired with a DeltaVision System microscope from Applied Precision.

Two experiments were chosen from the set available on the YRCPIR. The criteria used for choosing these experiments were to ensure that the images from these experiments had a significant presence of noise to illustrate the efficacy of the tested methods. A constraint placed on the choices however was that there had to be a contrast to noise ratio (CNR) of about 10 dB between the intracellular signal and background. The experiments chosen were experiment 80 and the first colour channel of experiment 165099. Both the colour channels of experiment 80 were chosen since they satisfied the criteria and constraints; however, only the first colour channel was selected for experiment 165099 because the other did not satisfy the CNR requirement. The experiment was chosen nonetheless due to the interesting intracellular fluorescing structure. The fluorescent protein and the number of colour channels acquired in each of these experiments are summarized in Table 1.

In the experiments considered above, only the colour channels were used for algorithm validation. The other channels typically present in the experiments are the FRET channels, which differentiate the signal between closely interacting molecules, and the differential interference contrast channel. The differential interference contrast and FRET channels were discarded because their image intensity distributions are not well-modelled by the Poisson-Gaussian mixture used here, and thus beyond the scope of this work.

### Competing Algorithms

We compared against state-of-the-art spatial algorithms for fluorescence microscopy noise reduction (Implementation sources of these algorithms are listed in the Supplementary Information). The algorithms compared against were:

**NCDF** Adaptive Complex Diffusion Despeckling Filter^34^.

**DiffusionO** Anisotropic Non-Linear Diffusion Imaging^13^.

**Molecular** Hybrid Model for Molecular Image Denoising^15^.

**MSVST** Multiscale Variance-Stabilizing Transform^9^.

**VWNF** Versatile Wavelet Domain Noise Filtration Technique^6^.

**PURELET** PURE-LET for Poisson Image denoising^7^.

Each algorithm has had their parameters trained for fluorescence microscopy image noise reduction, evaluated through quantitative metrics, for comparison. This process is outlined in the next subsection. The proposed algorithm will be referred to as SRF in figures and tables.

### Quantitative Analysis

We divided the quantitative analysis into two sections: synthetic and empirical. For the synthetic analysis, we evaluated each algorithm based on the signal-to-noise ratio (SNR), the improvement in signal-to-noise ratio (ISNR), and the peak signal-to-noise ratio (PSNR), between the noise-reduced image and the synthetic ground truth. The empirical analysis is done by comparing the SNR and CNR values of the noise-reduced images between tested methods as well as the original noisy images. This was done for each channel in the empirical experiments separately.

To improve fairness of the tests, all tested methods are configured using a grid search to achieve optimal PSNR on a subset of the synthetic data and used those same parameters on the remaining synthetic data. The empirical data from the YRCPIR had the parameters optimized for each tested method using a grid search to maximize SNR.

To evaluate the effectiveness of the algorithm on synthetic data, we used the SNR, ISNR and PSNR. The SNR provides a decibel representation of the ratio between the squared sum of the signal and the mean squared error (MSE) between the noisy and noise reduced image, Eq. (22). The ISNR value provides a decibel representation of the ratio between the MSE between the noisy image and the ground truth and the MSE between the noisy and noise reduced image, Eq. (23). The PSNR provides a decibel representation of the ratio between the maximum possible data value over the MSE between the noise reduced image and the ground truth, Eq. (24). The higher any of these metrics, the closer the denoised image is to the ground truth.


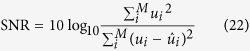



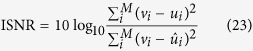



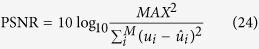


where *MAX* is the maximum possible value.

In addition to looking at image quality metrics, we will look at the computational cost of each algorithm as it processes the synthetic images. The average computational cost in seconds over all the photon-conversion factors will be summarised in a table with the lowest value performing the best. The methods were run in MATLAB 2014b (Mathworks, Inc., MA) with an Intel i5-4210M CPU with 8 GB of RAM.

**Figure 5:**
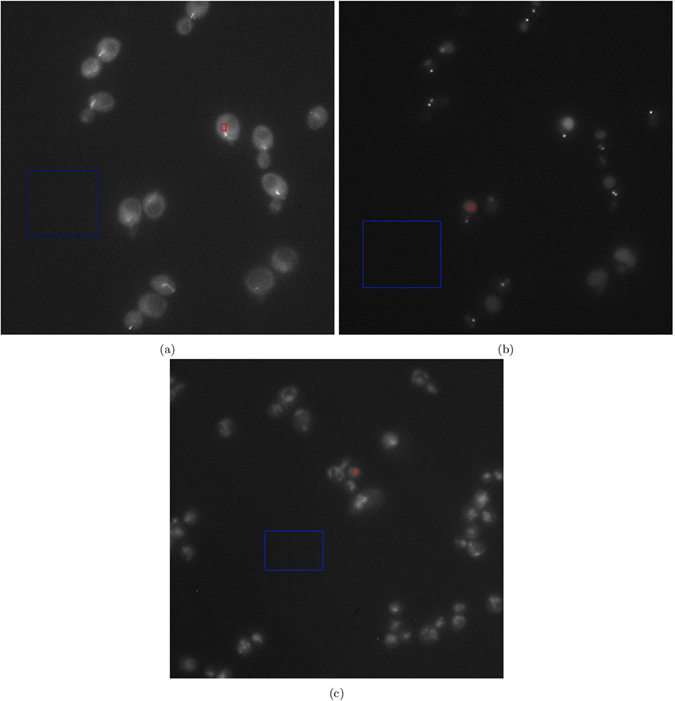
Regions used for calculating SNR and CNR for the empirical experiments. (**a**) Experiment 80: green channel, (**b**) experiment 80: red channel, and (**c**) experiment 165099: blue channel. The red bounding box represents a assumed smooth cell signal region and the blue bounding box is a region of assumed smooth background.

For quantitative analysis of the compared approaches on empirical data, there is no ground truth, so we use the Gaussian representation of SNR and CNR, Eq. (25) and Eq. (26), respectively. These metrics were calculated for two selected regions. One region was chosen from the background while the other was selected in a region of homogeneous intensity in a cell. The background region was representative of the underlying noise process while the cell region was selected as representative of the signal. For both the experiments, the regions chosen are shown in Fig. 5. SNR was calculated as follows, in decibels:


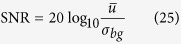


where 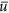 defines the mean value of the cell region and *σ* defines the standard deviation of the background region. CNR is also expressed in decibels as:


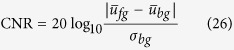


where 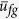 and 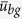 are the mean values of the selected cell region and the background region, respectively, and *σ*_*bg*_ is the standard deviation of the background region.

## Results

We present the quantitative results of the synthetic and empirical experiments comparing the proposed algorithm to the other competing approaches in this section.

**Figure 6:**
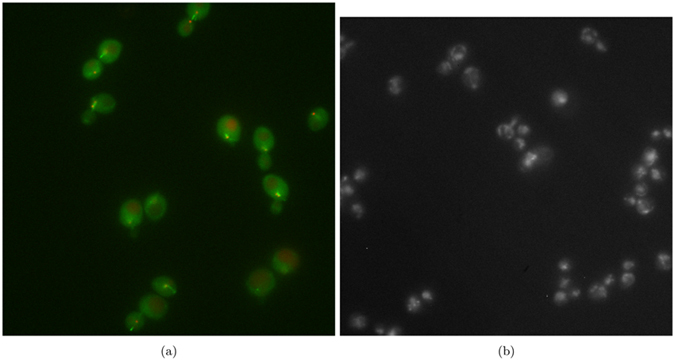
Baseline images used for empirical parameter tuning. (**a**) Experiment 80 baseline multi-channel image, and (**b**) experiment 165099 baseline multi-channel image.

Figure 4 shows the phantom image used for parameter tuning of the SRF and other competing algorithms for the synthetic testing. The baseline images in Fig. 6 were used for algorithm parameter tuning for empirical testing.

### Synthetic Results

The results for the synthetic experiments are summarised in Tables 2, 3, 4. Due to the stochastic nature of the proposed algorithm, it was run 30 times and the average metrics are presented.

The SNR, ISNR, and PSNR results are summarized in Tables 2, 3, 4. SRF produces the best overall SNR, ISNR, and PSNR values with photon-conversion factors of *β* = 0.2 ···0.9 with the VWNF method producing the next highest.

**Figure 7:**
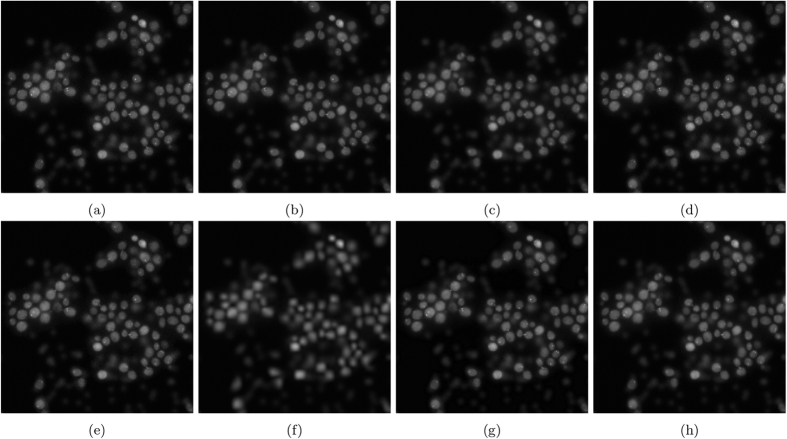
Noise reduction results for the noise-contaminated synthetic phantom having a photon-conversion factor (*β*) of 0.5. (**a**) Baseline (PSNR: 31.32 dB), (**b**) SRF (PSNR: 38.26 dB), (**c**) NCDF (PSNR: 24.95 dB), (**d**) DiffusionO (PSNR: 32.17 dB), (**e**) Molecular (PSNR: 29.92 dB), (**f**) MSVST (PSNR: 28.70 dB), (**g**) VWNF (PSNR: 33.94 dB), and (**h**) PURELET (PSNR: 27.99 dB).

**Figure 8:**
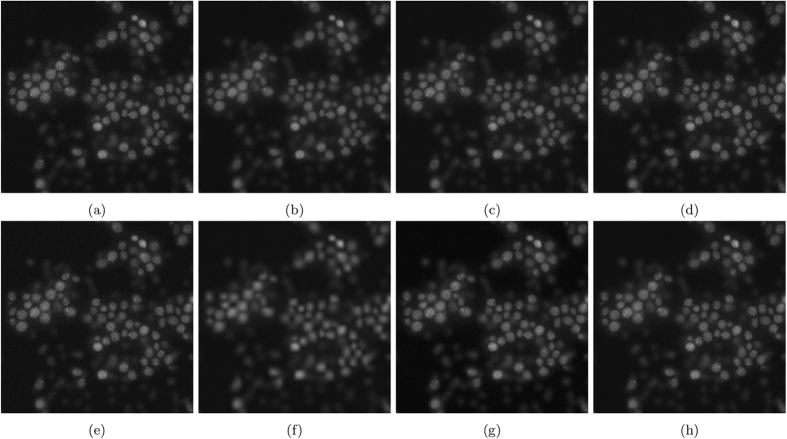
Noise reduction results for the noise-contaminated synthetic phantom having a photon-conversion factor (*β*) of 0.1. (**a**) Baseline (PSNR: 22.64 dB), (**b**) SRF (PSNR: 27.14 dB), (**c**) NCDF (PSNR: 19.03 dB), (**d**) DiffusionO (PSNR: 22.99 dB), (**e**) Molecular (PSNR: 19.54 dB), (**f**) MSVST (PSNR: 29.11 dB), (**g**) VWNF (PSNR: 31.12 dB), and (**h**) PURELET (PSNR: 23.61 dB).

**Figure 9:**
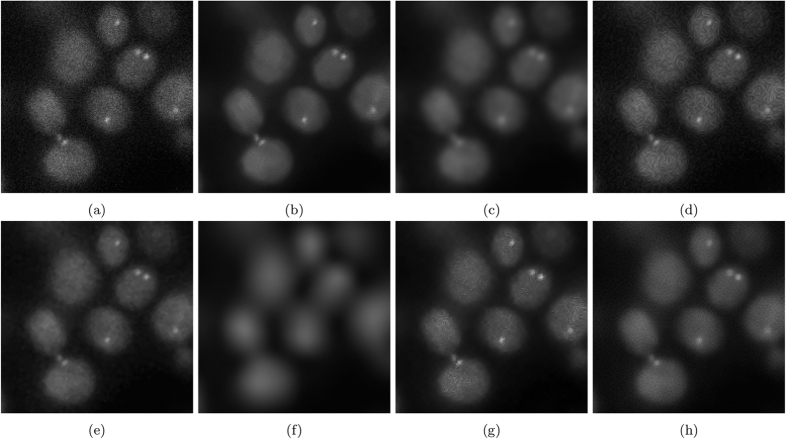
Magnified noise reduction results for the noise-contaminated synthetic phantom having a photon-conversion factor (*β*) of 0.5. (**a**) Baseline, (**b**) SRF, (**c**) NCDF, (**d**) DiffusionO, (**e**) Molecular, (**f**) MSVST, (**g**) VWNF, and (**h**) PURELET.

**Figure 10:**
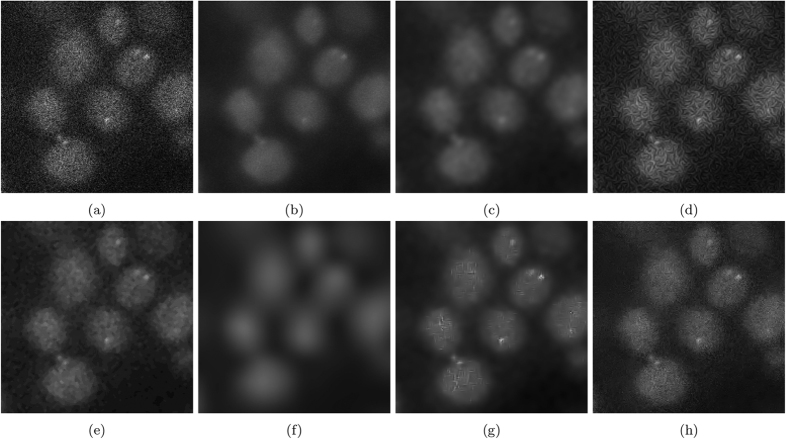
Magnified noise reduction results for the noise-contaminated synthetic phantom having a photon-conversion factor (*β*) of 0.1. (**a**) Baseline, (**b**) SRF, (**c**) NCDF, (**d**) DiffusionO, (**e**) Molecular, (**f**) MSVST, (**g**) VWNF, and (**h**) PURELET.

The resulting images are shown in Fig. 7. Figures 7 and 8 show the results of photon-conversion factor *β* = 0.5 and *β* = 0.1. Figures 9 and 10 show a magnified region showing a group of cells from Figs 7 and 8, respectively.

In Fig. 7, which is representative of a common photon starved case with a photon conversion factor of *β* = 0.5, the proposed algorithm is able to maintain the structural edges of the cells while smoothing inter-cellular intensity inconsistencies, noise, and maintaining low frequency intensity changes to achieve the highest SNR, ISNR, and PSNR. Other algorithms either lose cellular structure to reduce inter-cellular noise like NCDF and, in a more extreme case, MSVST, or maintain cellular structure at the expense of reducing noise like DiffusionO and VWNF.

In the highly photon starved case with a photon conversion factor of *β* = 0.1, the method that resulted in the highest metrics in Fig. 8 was VWNF followed by MSVST. This is the conversion factor that where SRF ranked third amongst all the algorithms. The results of the SRF algorithm show good edge structure preservation compared to NCDF, Molecular, and MSVST, and improved noise suppression compared to the baseline, DiffusionO, Molecular, VWNF, and PURELET. However, there is some loss in smaller details in the results of the SRF algorithm (such as the loss of a small nucleoli in the right-most cell (see Fig. 10), which illustrates limitations with the use of the proposed SRF algorithm under highly photon starved cases. Note that while VWNF was able to achieve a higher SNR, ISNR, and PSNR, intra-cellular noise was not reduced, and wavelet-related artefacts were observed within the cell structure.

In Table 5, the computational cost of each algorithm is summarised. The SRF algorithm does take longer to process each image relative to the state of art, but only half as long as MSVST. In the other metrics, it is seen that SRF, while taking half as long, can still perform comparably to MSVST.

### Empirical Results

**Figure 11:**
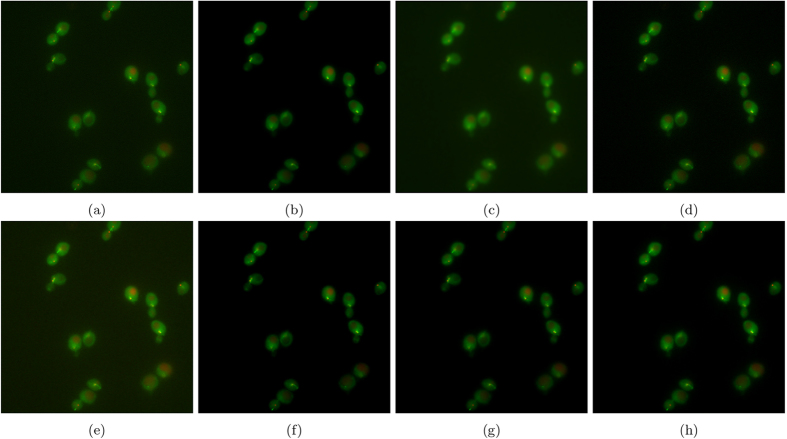
The resultant noise-reduced images for experiment 80. (**a**) Baseline, (**b**) SRF, (**c**) NCDF, (**d**) DiffusionO, (**e**) Molecular, (**f**) MSVST, (**g**) VWNF, and (**h**) PURELET.

**Figure 12:**
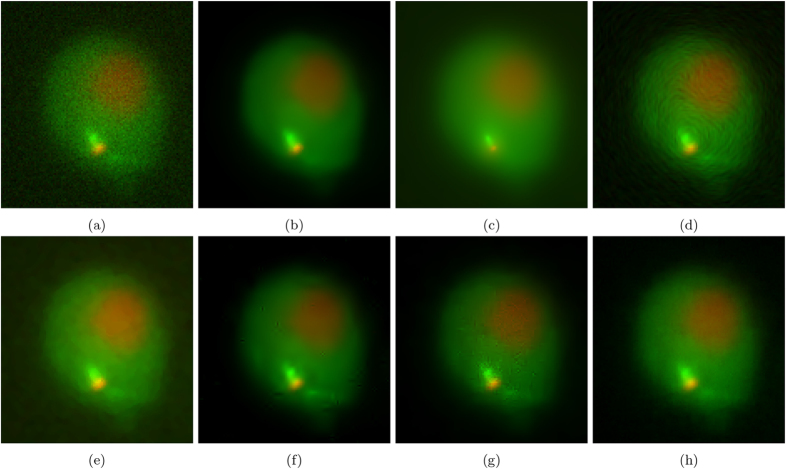
The resultant noise-reduced images for experiment 80. These images focus on a single cell in experiment 80. (**a**) Baseline, (**b**) SRF, (**c**) NCDF, (**d**) DiffusionO, (**e**) Molecular, (**f**) MSVST, (**g**) VWNF, and (**h**) PURELET.

**Figure 13:**
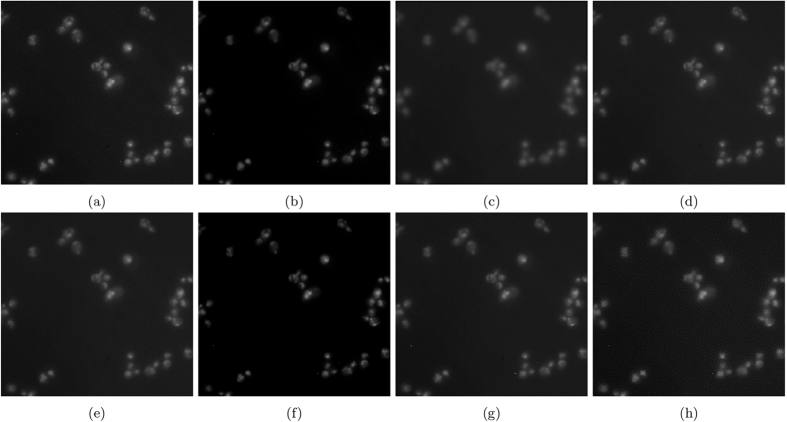
The resultant noise-reduced images for experiment 165099. (**a**) Baseline, (**b**) SRF, (**c**) NCDF, (**d**) DiffusionO, (**e**) Molecular, (**f**) MSVST, (**g**) VWNF, and (**h**) PURELET.

**Figure 14:**
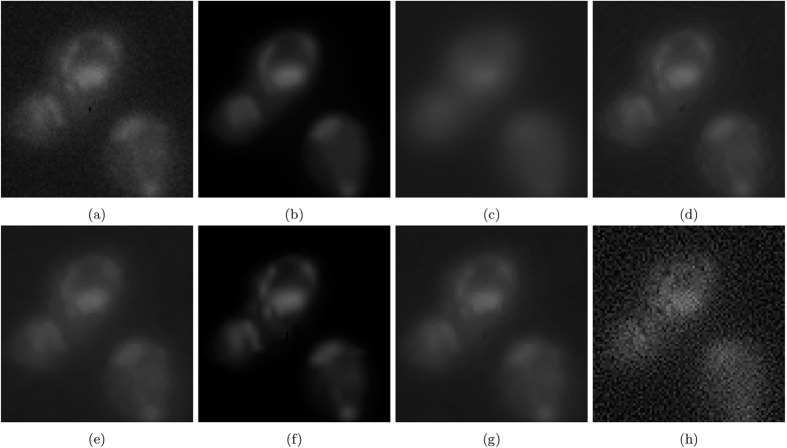
The resultant noise-reduced images for experiment 165099. These images focus on a group of cells in experiment 165099. (**a**) Baseline, (**b**) SRF, (**c**) NCDF, (**d**) DiffusionO, (**e**) Molecular, (**f**) MSVST, (**g**) VWNF, and (**h**) PURELET.

Figure 11 shows the results of experiment 80, with Fig. 12 magnifying a group of cells for observation. Figure 13 shows the results of experiment 165099, with Fig. 14 magnifying a group of cells for observation. The noise reduction results on the empirical images are presented in their merged representation where the colour of the emission wavelengths are merged into a full colour representation (ie. the red channel is represented by the colour red in this representation and similarly for the blue and green channel, if they exist). SNR and CNR results are presented in Table 6 and 7. Simlar to the synthetic testing, due to the stochastic nature of the algorithm, SRF was run 30 times and the average SNR and CNR results were measured.

The proposed algorithm achieves SNR values competitive to the next highest performing method, NCDF. SRF produced the highest SNR in the red channel of experiment 80 while producing the second highest in the green channel of experiment 80. The CNR performance of SRF shows to be the highest across both channels in experiment 80. Looking at the results of experiment 80 in Fig. 12, maintaining the structure of the cell and preserving fine features while reducing noise is not something all the algorithms were capable of. MSVST and NCDF reduced noise but sacrificed the detail of the bright fluorescent features. VWNF was able to preserve the edge structure and was able to reduce noise but produced wavelet artefacts. SRF was able to reduce background and intra-cellular noise while maintaining structure and fine details.

In experiment 165099, SRF produces competitive SNR and CNR results against the higher performing algorithms by achieving the next highest results. In Fig. 14, the groups of cells are not entirely above the noise-floor and most algorithms found it difficult to recover a noise-free image while being able to maintain the separability of the cells and their internal structure while reducing noise, simultaneously. VWNF and Molecular was able to do this quite well; Molecular did it too, but at the risk of heavy quantization. DiffusionO was able to maintain internal cellular structure, at the risk of presuming structure where none presumably exist. SRF provided noise reduction while maintaining separability in the cells and preserving the edge structure of the cell in the bottom right corner.

## Discussion

Fluorescence microscopy is a useful tool in a biologist’s toolkit and through the use of the proposed SRF algorithm, we have demonstrated that improvements to SNR and CNR can be made. This improved clarity aids in observing cellular structure and inter and intra-cellular dynamics.

The capabilities of the SRF algorithm were demonstrated in this paper against other tested methods for fluorescence microscopy noise reduction using synthetic and empirical datasets. Empirical studies quantitatively demonstrated SRF’s competitiveness using SNR and CNR metrics, and synthetic tests showed similar results using the SNR, ISNR, and PSNR metric. A key contributing factor to SRF’s performance in noise reduction in the common photon starved cases is that, by combining random graph and field theory into a unified random field modelling framework, the proposed SRF algorithm can better account for abrupt data uncertainties in the common photon starved cases while preserving cellular structures in the noise reduction process. However, the SRF algorithm shows limitations when dealing with highly photon starved cases (see Fig. 10) where some loss in smaller details is noticeable and is noteworthy as an area to improve in future work.

An interesting area worth exploring in the future is in the way the stochastic edge connectivity is established in the SRF model. In the current realization of the SRF model, the stochastic edges aim to establish weights within a local neighborhood surrounding a pixel region in the random field, under an assumption of local spatial-feature smoothness, for noise reduction. However, in fluorescence microscopy, many cells can be imaged at the same time to study multiple fluorophore responses, but the cells are usually sparsely distributed across the entire image. As such, a shortcoming of the current realization of the SRF model is that it is limited in making use of the additional information contained by sparsely distributed cells beyond the local neighborhood, thereby potentially limiting noise reduction performance. One can potentially take advantage of the similar fluorophore responses of such sparsely distributed cells, for the purpose of noise reduction, in a manner that is similar to non-local noise reduction methods^16^,^17^. As such, we aim to extend the SRF model by exploring new strategies to better incorporate long-range stochastic edge connectivity into the model while maintaining computational efficiency. Another research direction that is worth investigating is the extension of the proposed method to take into account the point spread function (PSF) of the microscope to provide simultaneous noise reduction and deconvolution, which can have significant benefits in improving image quality for fluorescence microscopy.

## Additional Information

**How to cite this article**: Haider, S. A. *et al.* Fluorescence microscopy image noise reduction using a stochastically-connected random field model. *Sci. Rep.* **6**, 20640; doi: 10.1038/srep20640 (2016).

## Supplementary Material

Supplementary Information

## Figures and Tables

**Table 1:** Properties of Validation Datasets.

Experiment #	Fluorescent Protein	Colour Channels
80	KIP3 (GFP)	Green, Red
	NUF2 (CHERRY)	
165099	Nil	Blue

Summaries of the experiment number, fluorescent proteins and colour channels for the experiments used for validation experiments.

**Table 2:** SNR Results for synthetic experiment.

Method	SNR(dB)								
	*β* = 0.1	*β* = 0.2	*β* = 0.3	*β* = 0.4	*β* = 0.5	*β* = 0.6	*β* = 0.7	*β* = 0.8	*β* = 0.9
Baseline	2.61	3.70	4.88	6.13	7.40	8.53	9.25	9.31	8.67
SRF	7.60	**17.00**	**19.72**	**16.80**	**20.34**	**18.85**	**18.05**	**18.47**	**18.11**
NCDF	1.11	2.78	5.73	6.15	7.03	7.96	6.85	7.74	8.44
DiffusionO	7.53	7.53	10.42	10.20	14.25	15.51	14.39	14.83	16.02
Molecular	4.03	4.03	7.16	9.07	12.00	10.97	11.46	10.56	10.45
MSVST	10.68	10.68	10.50	10.78	10.56	10.97	10.48	10.56	10.64
VWNF	13.20	15.55	15.19	14.25	16.02	16.30	16.74	15.99	17.14
PURELET	5.69	6.99	9.70	9.15	10.07	12.23	10.65	9.09	11.00

Using a photon-conversion factor of (*β*) 0.5, optimal parameters were found via grid search to produce the highest PSNR for all methods. This table summarizes the SNR, using Eq. 22, values of those parameters applied for the remaining photo-conversion factors. Highest values are shown in bold and the Baseline refers to the noisy synthetic phantom.

**Table 3:** ISNR Results for synthetic experiment.

Method	ISNR(dB)								
	*β* = 0.1	*β* = 0.2	*β* = 0.3	*β* = 0.4	*β* = 0.5	*β* = 0.6	*β* = 0.7	*β* = 0.8	*β* = 0.9
Baseline	0.00	0.00	0.00	0.00	0.00	0.00	0.00	0.00	0.00
SRF	4.99	**13.29**	**14.84**	**10.67**	**12.94**	**10.32**	**8.80**	**9.16**	**9.43**
NCDF	−1.50	−0.92	0.85	0.02	−0.37	−0.57	−2.41	−1.57	−0.24
DiffusionO	4.92	3.83	5.54	4.07	6.85	6.98	5.13	5.52	7.34
Molecular	1.42	0.33	2.28	2.94	4.60	2.44	2.20	1.25	1.77
MSVST	8.07	6.98	5.62	4.65	3.16	2.44	1.22	1.25	1.97
VWNF	10.59	11.85	10.31	8.12	8.62	7.77	7.48	6.68	8.47
PURELET	3.08	3.29	4.82	3.02	2.67	3.70	1.39	−0.22	2.32

Using a photon-conversion factor of (*β*) 0.5, optimal parameters were found via grid search to produce the highest PSNR, using Eq. 23, for all methods. This table summarizes the ISNR values of those parameters applied for the remaining photo-conversion factors. Highest values are shown in bold and the Baseline refers to the noisy synthetic phantom.

**Table 4:** PSNR Results for synthetic experiment.

Method	PSNR(dB)								
	*β* = 0.1	*β* = 0.2	*β* = 0.3	*β* = 0.4	*β* = 0.5	*β* = 0.6	*β* = 0.7	*β* = 0.8	*β* = 0.9
Baseline	22.64	24.11	28.64	28.91	31.32	32.19	31.97	33.18	34.17
SRF	25.52	**34.91**	**37.64**	**34.72**	**38.26**	**36.77**	**35.97**	**36.39**	**36.03**
NCDF	19.03	20.70	23.65	24.07	24.95	25.88	24.77	25.66	26.36
DiffusionO	25.45	25.45	28.34	28.12	32.17	33.43	32.31	32.75	33.94
Molecular	21.95	21.95	25.08	26.99	29.92	28.89	29.38	28.48	28.37
MSVST	28.60	28.60	28.42	28.70	28.48	28.89	28.40	28.48	28.56
VWNF	31.12	33.47	33.11	32.17	33.94	34.22	34.66	33.91	35.06
PURELET	23.61	24.91	27.62	27.07	27.99	30.15	28.57	27.01	28.92

Using a photon-conversion factor of (*β*) 0.5, optimal parameters were found via grid search to produce the highest PSNR, using Eq. 24, for all methods. This table summarizes the PSNR values of those parameters applied for the remaining photo-conversion factors. Highest values are shown in bold and the Baseline refers to the noisy synthetic phantom.

**Table 5:** Computational Cost for each algorithm.

Method	Time(s)
SRF	81.59
NCDF	14.19
DiffusionO	5.40
Molecular	8.85
MSVST	120.15
VWNF	11.25
PURELET	32.48

This table summarizes the computational cost of each method. Highest value is shown in bold.

**Table 6:** SNR values for empirical experiments.

Method	SNR (dB)		
	ID 80		ID 165099
	Green	Red	Blue
Baseline	8.27	9.63	15.15
SRF	14.91	**39.83**	20.77
NCDF	**15.18**	32.59	**21.87**
DiffusionO	12.28	18.86	19.50
Molecular	14.03	26.67	19.30
MSVST	13.43	30.57	18.97
VWNF	12.84	31.89	17.88
PURELET	13.95	35.34	5.88

Highest values are shown in bold. ID numbers denote the experiment ID of each dataset in^4^.

**Table 7:** CNR values for empirical experiments.

Method	CNR (dB)		
	ID 80		ID 165099
	Green	Red	Blue
Baseline	13.81	10.68	24.46
SRF	**119.85**	**92.41**	60.23
NCDF	118.73	85.28	44.32
DiffusionO	32.25	24.97	42.78
Molecular	55.35	43.42	52.43
MSVST	87.78	73.26	**80.93**
VWNF	88.86	67.40	51.18
PURELET	73.85	61.10	5.56

Highest values are shown in bold. ID numbers denote the experiment ID of each dataset in^4^.
