# Supplementary material for: Fluorescence microscopy image noise reduction using a stochastically-connected random field model
Source: Sci Rep. 2016 Feb 17;6:20640. doi: 10.1038/srep20640 (PMC4756687; doi:10.1038/srep20640)
Supplement: Supplementary Information [file srep20640-s1.pdf]

Supplementary Information for Fluorescence microscopy image noise reduction using a stochastically-connected random field model

S.A. Haider[1,*], A. Cameron[1], P. Siva [1], D. Lui [1], M.J. Shafiee [1], A. Boroomand [1], N. Haider [2], A. Wong [1]

1 Vision and Image Processing (VIP) Research Group, Department of Systems Design Engineering, University of Waterloo, 200 University Avenue W, Waterloo, ON, N2T 3G1, Canada

2 Department of Medical Biophysics, University of Toronto, 610 University Avenue, Toronto, ON, M5G 2M9, Canada

∗ E-mail: sa2haide@uwaterloo.ca

Implementations of the competing algorithms used for comparison are available at the websites mentioned here:

NCDF Adaptive Complex Diffusion Despeckling Filter. Source Code: `http://www.mathworks.com/matlabcentral/fileexchange/29801`.

DiffusionO Anisotropic Non-Linear Diffusion Imaging. Source Code, `http://www.mathworks.com/matlabcentral/fileexchange/25449`.

Molecular Hybrid Model for Molecular Image Denoising. Source Code, `http://www.mathworks.com/matlabcentral/fileexchange/21289`.

MSVST Multiscale Variance-Stabilizing Transform. Source Code, `https://sites.google.com/site/bowangzhang/software/msvst79.tar.gz`.

VWNF Versatile Wavelet Domain Noise Filtration Technique. Source Code, `http://telin.ugent.be/~sanja/Sanja_files/WaveletDEN.htm`.

PURELET PURE-LET for Poisson Image denoising. Source Code, `http://www.mathworks.com/matlabcentral/fileexchange/31557-pure-let-for-poisson-image-denoi`
